# Antiproliferative Activity and Induction of Apoptosis in PC-3 Cells by the Chalcone Cardamonin from *Campomanesia adamantium* (Myrtaceae) in a Bioactivity-Guided Study

**DOI:** 10.3390/molecules19021843

**Published:** 2014-02-07

**Authors:** Aislan Cristina Rheder Fagundes Pascoal, Carlos Augusto Ehrenfried, Begoña Gimenez-Cassina Lopez, Thiago Matos de Araujo, Vinicius D’ávila Bitencourt Pascoal, Rovilson Gilioli, Gabriel Forato Anhê, Ana Lúcia Tasca Goes Ruiz, João Ernesto de Carvalho, Maria Élida Alves Stefanello, Marcos José Salvador

**Affiliations:** 1 Graduate Program in Biosciences and Technology of Bioactive Products, Pharmacy course, State University of Campinas, Campinas, 6109, São Paulo 13083-970, Brazil; E-Mails: aislanfagundes@yahoo.com.br (A.C.R.F.P.); begogcl@gmail.com (B.G.-C.L.); 2 Department of Chemistry, Federal University of Paraná, 19081, Curitiba 81531-990, Paraná, Brazil; E-Mails: slash@ufpr.br (C.A.E.); elida@ufpr.br (M.E.A.S.); 3 Department of Pharmacology, Faculty of Medical Sciences, State University of Campinas, Campinas 13083-887, São Paulo, Brazil; E-Mails: thiagomatosaraujo@gmail.com (T.M.A.); anhegf@yahoo.com.br (G.F.A.); 4 Faculty of Basic Sciences, Fluminense Federal University, Nova Friburgo 24020-420, Rio de Janeiro, Brazil; E-Mail: vinicius_pascoal@yahoo.com.br; 5 Multidisciplinary Center for Biological Research, Laboratory Animal Quality Control, State University of Campinas, l6095, Campinas 13083-877, São Paulo, Brazil; E-Mail: rovilson@unicamp.br; 6 Division of Pharmacology and Toxicology, Multidisciplinary Center for Chemical, Biological and Agricultural, State University of Campinas, 6171, Campinas 13081-970, São Paulo, Brazil; E-Mails: aa_ruiz@hotmail.com (A.L.T.G.R.); carvalho_je@yahoo.com.br (J.E.C.)

**Keywords:** *Campomanesia adamantium*, Myrtaceae, chalcone, cardamonin, apoptosis, cancer

## Abstract

The Myrtaceae family is a common source of medicines used in the treatment of numerous diseases in South America. In Brazil, fruits of the *Campomanesia* species are widely used to make liqueurs, juices and sweets, whereas leaves are traditionally employed as a medicine for dysentery, stomach problems, diarrhea, cystitis and urethritis. Ethanol extracts of *Campomanesia adamantium* (Myrtaceae) leaves and fruits were evaluated against prostate cancer cells (PC-3). The compound (2*E*)-1-(2,4-dihydroxy-6-methoxyphenyl)-3-phenylprop-2-en-1-one, cardamonin) was isolated from ethanol extracts of C. *adamantium* leaves in a bioactivity-guided study and quantified by UPLC-MS/MS. *In vitro* studies showed that the isolated chalcone cardamonin inhibited prostate cancer cell proliferation and decreased the expression of NFkB1. Moreover, analysis by flow cytometry showed that this compound induced DNA fragmentation, suggesting an effect on apoptosis induction in the PC-3 cell line.

## 1. Introduction

Application of natural products with therapeutic properties is as old as human civilization. Plants represent the largest sources of active substances that can be used in medical therapy due to the large structural diversity that these metabolites exhibit, being perhaps the oldest source of medicines for man [1]. Drugs derived from natural products with antibacterial, anticoagulant, antiparasitic, immunosuppressive and anticancer activity are capable of treating 87% of categorized human diseases [2]. Among the 128 anticancer drugs released on the market between 1981 and 2010, 12 were natural products and 32 were derived from natural products [3]. Such data justifies work in the area of natural products, particularly in view of its importance in the search for new drugs in cancer therapy.

The Myrtaceae (121 genera, 3,800–5,800 spp) is one of the most important families in tropical forests. They consist of aromatic trees or shrubs, which frequently produce edible fruit. In the neotropics around 1,000 species of this family can be found. Several members are used in folk medicine, mainly as anti-diarrheic, antimicrobial, antioxidant, cleansing, antitumoral, anti-rheumatic, and anti-inflammatory agents, as well as to lower blood cholesterol. In addition, some fruits are eaten fresh or used to make juices, liqueurs and sweets very much appreciated by the population [4]. *Campomanesia adamantium* (Cambess.) O. Berg (Myrtaceae) is an edible fruit tree native to Brazil, very abundant in areas of the Cerrado in the Midwest region and the Atlantic Forest in the Southeast and South regions of Brazil [5]. In previous studies, the fruit extracts of *C. adamantium* collected in the Midwest of Brazil (State of Mato Grosso do Sul) were evaluated against the microorganism *Mycobacterium tuberculosis.* Ethyl acetate extracts and fractions have shown promising antibacterial activity [6] and extracts from the leaves of *C. adamantium* have been evaluated by the DPPH assay for antioxidant activity together with the isolation of five flavanones (7-hydroxy-5-methoxyflavanone, 7-hydroxy-5-methyl-6-methoxyflavanone, 5,7-dihydroxy-6-methylflavanone, 5,7-dihydroxyflavanone and 8-methyl 5,7-dihydroxy-6,8-dimethylflavanone) and four chalcones (2',4'-dihydroxy-6'-methoxy-chalcone, 2',4'-dihydroxy-6'-methoxy-5'-methylchalcone, 2',4'-dihydroxy-6'-methoxy-3'-methylchalcone and 2',6'-dihydroxy-4'-methoxy-3',5'-dimethylchalcone) [7]. Another specimen, growing in the South of Brazil, also showed antioxidant activity associated with the presence of chalcones and flavonoids, including isoquercitrin, quercitrin, myricetin and quercetin [8]. Ethyl acetate and aqueous extracts from *C. adamantium* showed antinociceptive and anti-inflammatory activity *in vivo* and quercetin, myricitrin and myricetin were identified as possible compounds responsible for the anti-inflammatory activity, since such action was observed with these compounds *in vitro* [9]. Polyphenols have been identified in *C. adamantium* [8,9] and several studies have demonstrated antiproliferative activity involving this class of compounds [10,11]. Such data have encouraged the search for compounds with antiproliferative activity in *C. adamantium.*

In this study, we evaluated the antiproliferative activities of extracts from fruits and leaves of *C. adamantium* in PC-3 prostate cancer cells as a part of a bioprospecting program for the discovery of antiproliferative agents against cancer cell lines in the Brazilian flora. The chalcone cardamonin was isolated through bioassay-guided fractionation and quantified in fruit and leaf extracts. The antiproliferative activity of cardamonin and its effect on apoptosis induction in PC-3 prostate cancer cells was also studied.

## 2. Results and Discussion

### 2.1. Extraction and Isolation

**Figure 1:**
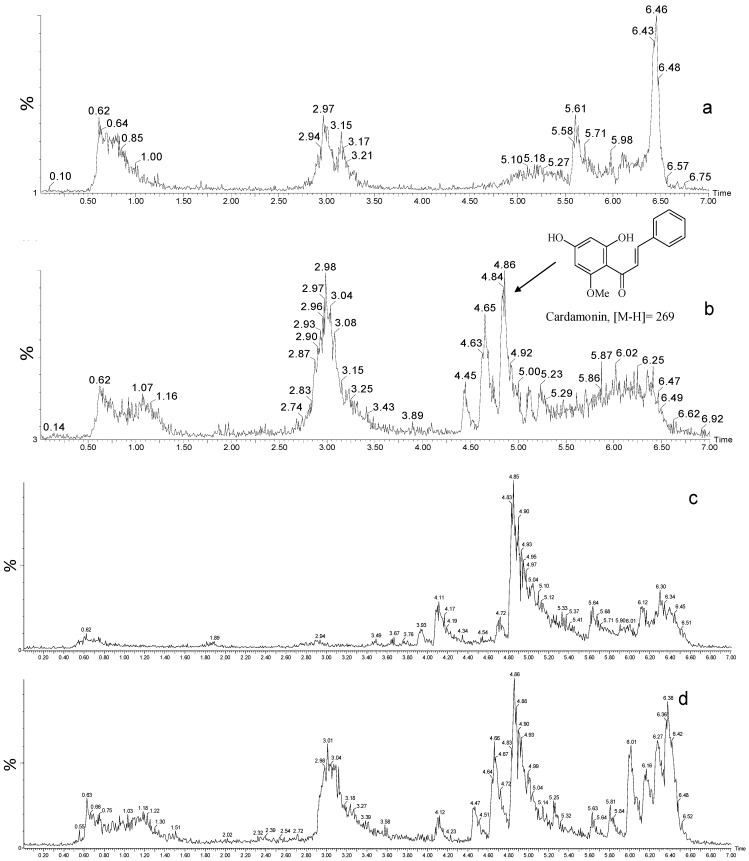
Chemical structure of cardamonin and chromatographic profile of extracts from leaves and fruits of *Campomanesia adamantium* by UPLC/MS/MS in negative ion mode. (**a**) fruit extract; (**b**) leaf extract; (**c**); fruit extract + standard sample of cardamonin (**d**) leaf extract+ standard sample of cardamonin.

From the crude ethanol extract of *C. adamantium* leaves we isolated the chalcone (2*E*)-1-(2,4-dihydroxy-6-methoxyphenyl)-3-phenylprop-2-en-1-one), denominated cardamonin (Figure 1). The ^1^H and ^13^C-NMR peaks were unambigously assigned by one-bond (HSQC) and long-range 1H-^13^C (HMBC) correlation experiments, and the molecular formula C_16_H_14_O_4_ was determined by MS ([M-H]^−^ 269). All spectral data are in agreement with literature data [12].

### 2.2. Quantification by UPLC-MS

UPLC–MS permitted the quantification of cardamonin concentrations in fruit and leaf extracts. Figure 1 shows chromatograms obtained from ethanol extracts of the fruits and leaves of *C. adamantium*. Quantification of the extract components highlighted cardamonin as the major compound. An average of 87.74 ± 1.07 μg/mg (mean ± standard deviation, analyzed in triplicate) was found in the leaf extract and 5.40 ± 13.07 μg/mg in the fruit extract. The linear fit was determined, with *R^2^* = 0.99.

### 2.3. Cardamonin, Fruit and Leaf Extracts Inhibited Survival of Cultured Human PC-3 Prostate Cancer Cells

**Figure 2:**
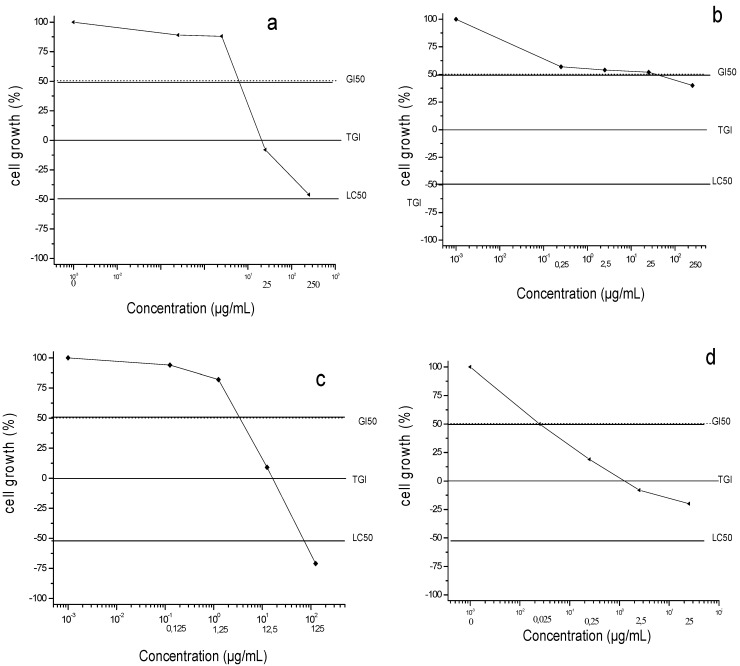
Antiproliferative activities in PC-3 cell line: (**a**) crude leaf extracts; (**b**) crude fruit extracts; (**c**) cardamonin and (**d**) doxorubicin after 48h treatment. G50: concentration necessary to inhibit 50% of growth; TGI: concentration necessary to inhibit 100% of growth and LC50 concentration necessary to be lethal 50% of cell line.

We studied the effects of fruit and leaf extracts and cardamonin on cell survival using human prostate cancer cell line PC-3 as a model, employing the sulforhodamine B assay to determine cell survival. This assay was used to determine the concentration necessary to inhibit 50% of growth (GI50) (Figure 2). The GI50 values for the leaf extracts, fruit extracts and cardamonin were 3.20, 14.25 and 11.35 μg/mL, respectively, compared to 0.028 μg/mL for the positive control doxorubicin. These results show a direct relationship between antiproliferative activity of extracts from fruits and leaves of *C. adamantium* and concentration of cardamonin. Many studies with different tumor cell lines and animal models have suggested that some chalcones can inhibit tumor initiation, tumor progression and induction of apoptosis [10,11,13].

### 2.4. Cardamonin Decreases NF-kB1 Expression Measured by qRT-PCR in Cultured PC-3 Cells

**Figure 3:**
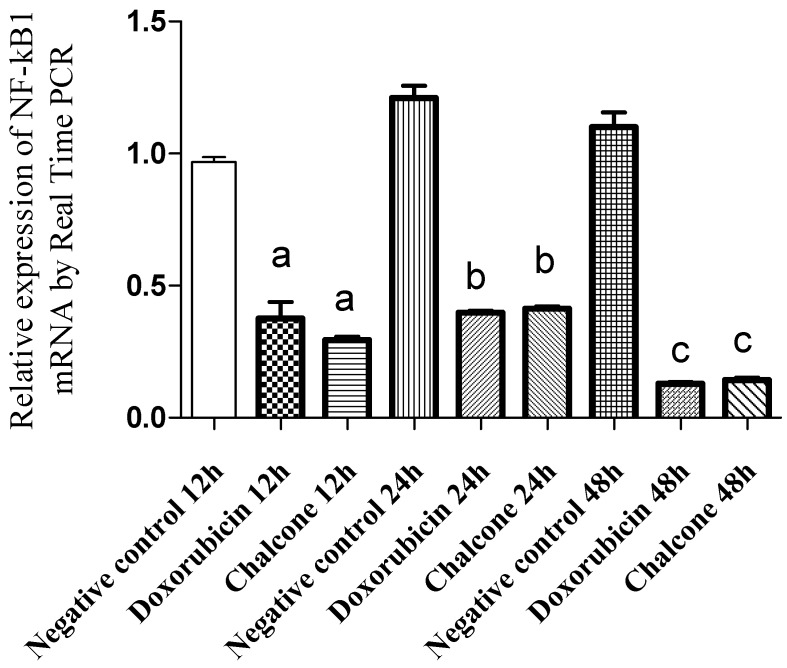
Relative expression of *NF-kB1* mRNA in PC-3 cells treated with cardamonin, measured by qRTPCR. Doxorubicin was used as a positive control. Values are the mean ± SD (*n* = 5). a = *p* < 0.05, ANOVA followed by the Tukey test, compared with the negative control at 12 h. b = *p* < 0.05, ANOVA followed by the Tukey test, compared with the negative control at 24 h. c = *p* < 0.05, ANOVA followed by the Tukey test, compared with the negative control at 48 h.

The NF-kB family consists of five proteins structurally related and functionally conserved: Rel A, Rel B, c-Rel, NFkB1 and NFkb2. The latter is a transcription factor that is activated in response to certain stimulations and plays a central role in the regulation of immune and inflammatory responses, apoptosis and oncogenesis [14,15]. It seems that NF-kB can convert inflammatory stimuli into tumor growth signals [16], suggesting it might be an important target for cancer therapy [14,15]. Cells treated with chalcone cardamonin and doxorubicin showed a decrease in gene expression NF-kB1 after 12, 24 and 48 h of treatment when compared to control cells grown in the presence of a diluent (Figure 3).

The decrease of *NF-kB1* expression in cells treated with the chalcone was 69.63% after 12 h of treatment and 65.87% within 24 h of treatment compared to the control cells. Similarly, for cells treated with doxorubicin, a decrease in gene expression of NF-kB1 was observed, diminishing 61.26% in 12 h of treatment and 67.11% within 24 h of treatment in comparison with control cells.

### 2.5. Flow Cytometric Analysis of DNA Fragmentation in Cultured Human PC-3 Prostate cancer Cells

**Figure 4:**
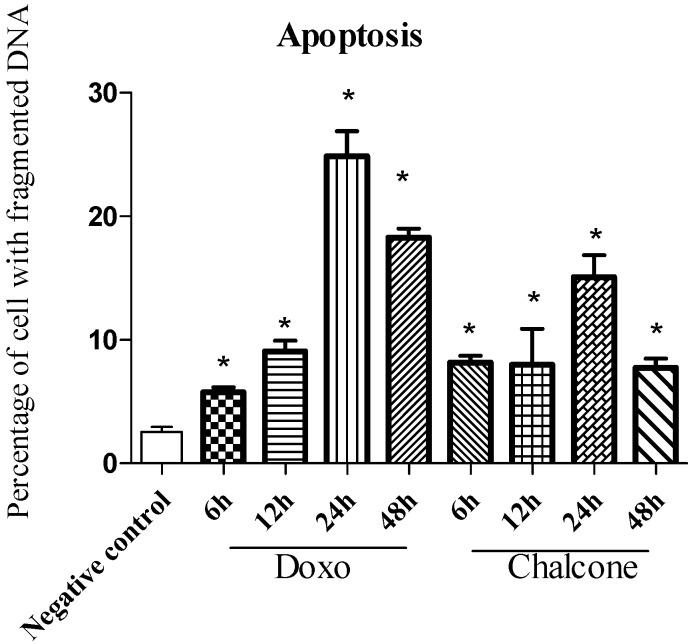
Percentage of PC-3cells with fragmented DNA as assessed by Flow Cytometry, indicating the end of the apoptosis process induced by treatment with the isolated cardamonin. Values are the mean ± SD (*n* = 5). * = *p* < 0.05, ANOVA followed by the Tukey test, compared with the negative control—cells cultived with diluent for 48 h.

**Figure 5:**
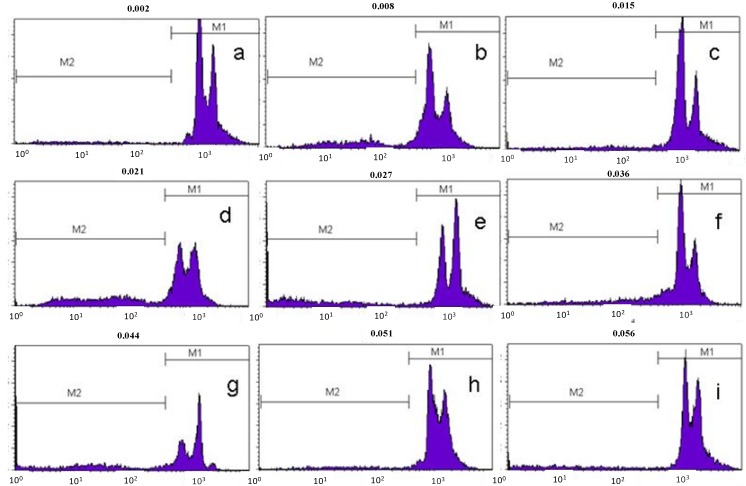
Histogram representation of DNA fragmentation in PC-3 cells in flow cytometry. M1 region corresponds to cells with intact DNA and M2 region corresponds to cells with fragmented DNA: (**a**)—negative control, cells treated with diluent for 48 h; (**b**)—cells treated with doxorubicin for 48 h; (**c**)—cells treated with chalcone for 48 h; (**d**)—cells treated with doxorubicin for 24 h; (**e**)—cells treated with chalcone for 24 h; (**f**)—cells treated with doxorubicin for 12 h; (**g**)—cells treated with chalcone for 12 h; (**h**)—cells treated with doxorubicin for 6h; (**i**)—cells treated with chalcone for 6 h.

The final stage of apoptosis is characterized by degradation of DNA with formation of final fragments of 180–190 nucleotide pairs [17]. PC-3 cells treated with the isolated chalcone cardamonin and doxorubicin were analyzed for DNA fragmentation after treatment for 6, 12, 24 and 48 h and compared to cells treated with the diluent for 48 h (Figure 4 and Figure 5). In cells treated for 6 h with cardamonin or with doxorubicin the percentage with fragmented DNA was 8.15% (±1.38) and 5.75% (±1.00), respectively.

However the highest percentage of fragmentation was observed after 24 h of treatment with 15.09% (±3.96) for chalcone and 24.86% (±4.93) for doxorubicin. There was a significant increase in apoptosis (*p* < 0.05) at all treatment times examined when compared to cells treated with the diluent. After 24 h there was a decline in the percentage of cells undergoing apoptosis, possibly due to metabolic inactivation of the drug present in the medium. These data support the conclusion that cardamonin, like doxorubicin, induces cell death by apoptosis in PC-3 cells.

Chalcones are intermediates in the biosynthesis of flavonoids and represent an important group of natural or synthetic compounds with an array of biological activities [18]. In cancer, several chalcones have been tested *in vitro* and some of them were cytotoxic [19]. In LNCaP prostate cancer cells, chalcones and dihydrochalcones were able to inhibit cell proliferation and induced apoptosis [20]. Antiproliferative activity studies with *Syzygium samarangense* (Myrtaceae) showed cytotoxic activity against the SW-480 human colon cancer cell line for natural chalcones, including cardamonin [21] and this compound suppresses the proliferation of colon cancer cells by promoting the degradation of intracellular *β*-catenin [22]. In another study it has been shown that cardamonin can block the nuclear factor-kappa B (NF-*κ*B) pathway in multiple myeloma [23].

Although the cardamonim concentration necessary to inhibit 50% of growth of PC-3 cell lines is higher than that for doxorubicin, this fact would be irrelevant if the new drug can circumvent problems encountered in cancer therapy regarding the high incidence of adverse effects such as high toxicity of the anticancer drugs or even development of multidrug-resistance (MDR) of certain types of tumors. New drugs may interact with different receptors and can be useful in the treatment of tumors that have developed multidrug resistance through cell drug efflux mechanisms, alteration in target enzymes expression and synthesis, changes in drug degradation or activation pathways, as well as failure of the apoptotic processes [24,25,26].

## 3. Experimental

### 3.1. General

NMR spectra were recorded on a Bruker AC 200 spectrometer (Bruker, Charlotte, NC, USA), observing ^1^H at 200 and ^13^C at 50, respectively. The solvent used was CDCl_3_ + MeOH-D_4_, with TMS as internal reference.

### 3.2. Plant Material

Leaves of *C. adamantium* Camb. (Myrtaceae) were collected in September 2008 and fruits in January 2010 in Curitiba, Paraná State, Brazil, (25°26'35''S, 49°14'58''W), and identified by Dr. Armando C. Cervi. A voucher specimen (UPCB 60503) was deposited at the herbarium of the Universidade Federal do Paraná (UFPR), Brazil.

### 3.3. Extraction and Isolation

For isolation of the active compound, dried and powdered leaves (270.3 g) were extracted at room temperature with hexane, followed by EtOH (3 × 1 L for each solvent). Solvents were removed under reduced pressure to yield the crude extracts. The ethanol extract (20 g) was dissolved in 50% aqueous EtOH (300 mL) and partitioned successively with hexane, CH_2_Cl_2_ and 1-butanol, (3 × 100 mL of each solvent), yielding the respective fractions (hexane—5.3 g, CH_2_Cl_2_—2.9 g, BuOH—3.74 g). The CH_2_Cl_2_ fraction was submitted to silica gel VLC eluted sequentially with CH_2_Cl_2_, EtOAc and MeOH (300 mL, each solvent). The fraction eluted with EtOAc (0.95 g) was submitted to silica gel CC and eluted sequentially with mixtures of EtOAc-hexane (1:1, 2:1, 5:1, 100 mL each), mixtures of EtOAc–MeOH (4:1, 2:1, 1:5, (100 mL each) and finally pure MeOH (50 mL). After TLC analysis, ten fractions were obtained. Fraction 2 (251.3 mg), eluted with EtOAc–hexane 1:1, was submitted to another CC, eluted with CH_2_Cl_2_, mixtures of CH_2_Cl_2_-MeOH (99:0.5, 99:1, 98:2, 50 mL each) and finally pure MeOH (20 mL) yielding seven subfractions after TLC analysis. The subfraction 2.2 (24.4 mg) was submitted to preparative TLC, eluted with hexane-acetone 3:2 (twice) to give the final isolated single compound (17.6 mg). 50g of dried and powdered fruits were extracted at room temperature with EtOH (3 × 150 mL) and the solvent removed under reduced pressure to give the crude extract. The spectral data of the isolated substance was: ^1^H-NMR (200 MHz, CDCl_3_ + MeOH-D_4_) δ_H_ (mult (*J* in Hz); H): 3.92 (*s*; 3H, 6'-OCH_3_), 5.97 (*d* (2.2); 1H, H-5'), 6.01 (*d* (2.2); 1H, H-3'), 7.41 (*m*; 3H, H-3, H-4 e H-5), 7.61 (*m*; 2H, H-2 e H-6), 7.75 (*d* (15,6); 1H, H-7), 7.91 (*d* (15.6); 1H, H-8). ^13^C-NMR (50 MHz, CDCl_3_ + MeOH-D_4_) δ_C_: 135.4 (C-1), 128.2 (C-2 e C-6), 128.7 (C-3 and C-5), 129.9 (C-4), 127.6 (C-8), 141.9 (C-7), 192.5 (C-9), 105.8 (C-1'), 1637.2 (C-2'), 96.1 (C-3'), 164.9 (C-4'), 91.2 (C-5'), 163.2 (C-6'), 55.7 (6'-OCH_3_).

### 3.4. UPLC-MS Quantification

Concentrations of cardamonin in fruit and leaf extracts were analyzed on an Acquity UPLC system (Waters, Milford, MA, USA) using a UPLC column (2.1 × 50 mm, 1.7 μm particle size) at a temperature of 30 °C. A gradient of (A) deionized purified water with 1% formic acid and (B) methanol (Tedia, Brazil) starting with 10% B and ramping to 100% B at 5 min, holding to 5.50 min, then returning to initial conditions and re-equilibrating at 7 min. Detection in negative ion modes was achieved on an Acquity TQD mass spectrometer (Micromass Waters, Milford, MA, USA) with capillary voltage—3,000 V, Cone—30 V, source temperature 150 °C; desolvation temperature 350 °C. A calibration curve was established by plotting the peak area against concentrations of standards in the range 100 ng/mL and 100 μg/mL with linear regression analysis. The limit of detection (LD) was visually evaluated with a signal-to-noise ratio of about 3:1 and limit of quantification (LQ) was defined as the lowest concentration on the standard curve. Previously, the ion of interest was subjected to MS/MS analysis to make sure that the *m/z* corresponded to the same compound as the standard by direct insertion mass spectrometry with electrospray ionization in negative mode (ESI-MS/MS).

### 3.5. Antiproliferative Assay

A prostate tumor cell line (PC-3), from the American Type Culture Collection (ATCC, Manassas, VA, USA), was used for assays. Briefly, the cells were distributed in 96-well plates (100 μL/well) and exposed to various concentrations of isolated chalcone cardamonin (0.25, 2.5, 25.0 and 125.0 μg/mL) in DMSO (0.1%) at 37 °C, with 5% of CO_2_, for 48 h. The final concentration of DMSO did not affect the cell viability. A 50% trichloroacetic acid solution was added and, after incubation for 30 min at 4 °C, the cells were washed with water and dried. Cell proliferation was determined by spectrophotometric quantification (at 540 nm) of the cellular protein content using sulforhodamine B [27]. The experiments were carried out at least in triplicate. Using the concentration-response curve for each cell line, TGI (concentration that produces total growth inhibition or cytostatic effect) was determined through non-linear regression (sigmoidal fitting) analysis using the software ORIGIN 8.0 (OriginLab Corporation, Northampton, MA, USA) [28]. Doxorubicin was used as positive control.

### 3.6. Analysis of NF-kB1 Expression by qRT-PCR

PC-3 cells were seeded in 6-well plates (10^5^ cells/well) and subjected to treatment with cardamonin (20 µg/mL) and doxorubicin (20 µg/mL) for 12 and 24 h. Cells treated with the diluent RPMI/DMSO in the ratio 95:5 were used as negative control. In order to analyze the effects of treatment on the expression of *NF-kB1*, qRT-PCR reactions were performed for each well of PC-3 cell after 12 h and 24 h of incubation. Total RNA was extracted with TRIzol^®^, according to the manufacturer’s recommendations. Reverse transcription was performed with 1 µg of RNA using the SuperScript III (Invitrogen, according to manufacturer’s instructions) and quantitative RT-PCR was performed using a 7500 System (Applied Biosystems, Carlsbad, CA, USA). PCR conditions (20 μL reaction volume) were: 50 °C for 2 min (1 cycle); 95 °C for 10 min (1 cycle); 95 °C for 15 s, 60 °C for 1 min (40 cycles). NF-kB1 primers were designed by Applied Biosystems (assay ID number: Hs00765730_m1) and endogenous control was GAPDH (VIC/MGB, Applied Biosystems).

### 3.7. DNA Fragmentation Analysis by Flow Cytometry

PC-3 cells were seeded in 6-well plates (10^5^ cells/well) and subjected to treatment with isolated chalcone cardamonin (20 µg/mL) and doxorubicin (20 µg/mL) for 6, 12, 24 and 48 h. Cells treated with the diluent RPMI/DMSO in the ratio 95:5 were used as negative control. After these procedures, the cells were removed from the wells using a scraper, washed with buffer, and resuspended in 300 µL of hypotonic PI solution containing propidium iodide (50 mg/mL 0.1% sodium citrate and 0.1% Triton X-100). Samples were incubated overnight at 4 °C and analyzed by flow cytometry in a Facs Scalibur system (BD Bioscience, San Jose, CA, USA). The histograms of the samples were analyzed using the Cell Quest Pro software [29].

### 3.8. Statistical Analysis

Data were expressed as means ± standard errors. Statistical significance of difference in measured variables between control and treated groups was determined by ANOVA followed by Tukey’s multiple comparison tests. Differences were considered significant at *p < 0.05*.

## 4. Conclusions

Based on our results, the compound cardamonin isolated from leaves of *C. adamantium* showed antiproliferative activity in the PC-3 cell line in a bioactivity-guided study, and induced apoptosis and downregulated the NFkB1 gene. Further studies investigating the role of the chalcone and *C. adamantium* natural products in biological systems are necessary to better define their therapeutic potential as a cytotoxic agent useful for possible *in vivo* applications in cancer treatment and prevention. Overall, our results may contribute to a comprehensive knowledge of the biological properties of *C. adamantium*, an edible fruit tree with medicinal properties native to Brazil, and furnish, at least in part, a scientific foundation to its popular use.
